# The pathophysiology of motor fatigue and fatigability in multiple sclerosis

**DOI:** 10.3389/fneur.2022.891415

**Published:** 2022-07-27

**Authors:** Robert Patejdl, Uwe K. Zettl

**Affiliations:** ^1^Oscar Langendorff Institute of Physiology, Rostock University Medical Center, Rostock, Germany; ^2^Department of Neurology, Clinical Neuroimmunology Section, Rostock University Medical Center, Rostock, Germany

**Keywords:** multiple sclerosis, motor fatigue, aerobic capacity, detraining, autonomic dysfunction

## Abstract

Multiple Sclerosis (MS) is a heterogeneous immune mediated disease of the central nervous system (CNS). Fatigue is one of the most common and disabling symptom of MS. It interferes with daily activities on the level of cognition and motor endurance. Motor fatigue can either result from lesions in cortical networks or motor pathways (“primary fatigue”) or it may be a consequence of detraining with subsequent adaptions of muscle and autonomic function. Programmed exercise interventions are used frequently to increase physical fitness in MS-patients. Studies investigating the effects of training on aerobic capacity, objective endurance and perceived fatigability have yielded heterogenous results, most likely due to the heterogeneity of interventions and patients, but probably also due to the non-uniform pathophysiology of fatigability among MS-patients. The aim of this review is to summarize the current knowledge on the pathophysiology of motor fatigability with special reference to the basic exercise physiology that underlies our understanding of both pathogenesis and treatment interventions.

## Introduction: Fatigue in multiple sclerosis

Multiple sclerosis (MS) is a clinically heterogeneous condition, often referred to as “a disease with a thousand different faces” (1, 2). Patients suffering from MS often experience a multitude of symptoms throughout their lifetime. Whereas, motor deficits are prominent and dominate both the social perspective on patient's disease and the clinically fundamental Expanded Disability Status Scale (EDSS), other symptoms are less easily accessible but nevertheless may have great impact on patients' quality of life and their self-reliance (3–8).

One of the most challenging non-motor complications of MS is the symptom complex termed “MS-fatigue” (1, 2, 9). It is frequent, occurring in a majority of MS patients at some point of disease (10, 11). And it is often hard to measure or even to define in individual patients, since it often occurs with comorbidities like depression or cognitive impairment and may be mimicked or overlayed with side effects of medications given for other MS-symptoms as spasticity of pain (12, 13).

Commonly, fatigue is classified as “primary fatigue” if it is considered to be the immediate result of immune-mediated damage to central nervous system (e.g., cortical lesions or lesions in the subcortical ascending arousal systems). In contrast, “secondary fatigue” results from factors that are indirectly related to MS, e.g., sleep disturbances, chronic urinary tract infections, the already mentioned pharmacological side effects or by deconditioning due to reduced physical activity levels (1).

A common distinction in studies on the pathophysiology of fatigue in patients with MS is made between “motor fatigue” and “cognitive fatigue” (9). Quantitative assessment instruments [e.g., the Modified Fatigue Impact Scale (14) or the Fatigue Scale for Motor and Cognitive Functions (15)] have been developed to differentiate fatigue and to facilitate future studies on the etiology and treatment responses of fatigue subtypes. However, a study analyzing questionnaires which were supposed to reflect the respective dimensions of MS-fatigue failed to confirm the assumed factor structure of three widely applied scales (16).

Regarding the clinical appearance of fatigue, three distinct prototypical manifestations of MS-fatigue have been deduced from pathophysiological considerations by Iriarte et al. (17): First, general adynamia or asthenia might result from inflammation, analogous to the well-known “cytokine induced sickness behavior” seen in the acute stage of many infectious diseases (18). Second, the long-known Uhthoff-phenomenon, a worsening of symptoms triggered by patients' engagement in physical activities, may be attributed to impaired action potential conduction in demyelinated axons that occurs with increased temperature (19, 20). Third, pathological mental and physical exhaustibility may occur independently of body temperature due to lesions in neuronal networks which reduce their functional efficiency and perseverance in task handling (21, 22).

Considering the concepts of primary and secondary MS-fatigue, it seems likely that both central and peripheral alterations are relevant in the pathophysiology of physical exhaustibility and generalized “motor fatigue.”

An assessment of the central component of fatigue is especially challenging due the intrinsic physiological complexity of CNS network function and the dependence on indirect readouts to analyze it. From the multitude of potential factors, three are of special relevance in the context of this review:

First, given the high incidence of depression and other mood disorders in MS, it is difficult to distinguish their genuine impact on the course and characteristics of reported fatigue (2, 23, 24). In imaging studies, lesions in specific brain areas were correlated with depression and fatigue in MS, suggesting a common elements in their pathophysiology (25, 26). It is nevertheless possible to define specific characteristics of concomitant depression and fatigue in MS patients on the basis of a parallel assessment of perceived “action control” (27). Despite the frequent coincidence of both symptoms in MS, there is no convincing data to support specific beneficial effects of antidepressant medications on MS-fatigue (28, 29).

Second, both increased and decreased connectivity between brain regions may give rise to motor fatigue. Functional magnetic resonance imaging (fMRI) studies could demonstrate that functional connectivity between brain regions is increased, although structural connectivity is decreased in patients with MS with cognitive deficits. Changes in functional connectivity may thus be maladaptive and lead to functional deficits even beyond isolated reduced performance in neuropsychological tasks (30). A transcortical magnetic stimulation study in RRMS–patients found an attenuated connectivity between premotor- and primary motorcortex which was significantly correlated with reported motor fatigue. In contrast, corticospinal connectivity was retained (31).

Third, a reduced or non-stable volitional drive to descending motor pathways will impede performance in motor tasks. Volitional drive is usually upregulated over time to keep constant force despite peripheral muscle fatigue in persistent submaximal contractions (32). With ongoing effort and exhaustion, feedback signals from peripheral muscles increase and make it more difficult to maintain volitional motor drive. Since MS-patients frequently suffer from depression, emotional stress and chronic pain, it seems justified to assume that their abilities to keep up adequate motor drive are reduced when compared to healthy controls (33, 34). Although the conduction pathways between brain and spinal cord are stable in MS-patients (35), a rundown in the actual motor output is supported by studies on central fatigue (36, 37).

Nevertheless, even with regular cortical network function and volitional drive, an important prerequisite for physical performance and endurance is an appropriate oxygen and energy supply which is physiologically adapted rapidly by appropriate changes of cardiac, pulmonary and vascular function parameters (38). Furthermore, effective movements rely on an accurate orchestration of motor units which is a complex computational task for the CNS (39). Finally, the muscle fibers themselves differ in their size, contractility and metabolism with respect to their utilization, i.e., training level (40, 41).

Considering this complex integration of peripheral and central factors, the intention of this review is to summarize our current knowledge on the interdependent pathophysiology of motor fatigue, fatigability and changes of physiological exercise responses in MS-patients.

## Current concepts of motor fatigue in MS: Definitions, assessment, pathophysiology and training interventions

In this section, we will discuss the existing knowledge and concepts of motor fatigue and fatigability in MS with a special focus on its pathophysiology. To avoid ambiguity, we will briefly discuss their definitions and operationalization first.

### Basic definitions of motor fatigue and fatigability in MS

By definition, the individual perception of being exhausted is purely subjective. In contrast, observable changes motor task performance can rather easily be detected and quantified. Therefore, the term “objective fatigability” can be used to address motor symptoms of MS-fatigue more specifically (42). On the other hand, the objective changes in motor functions may not fully reflect the degree of subjective impairment. Therefore, data from questionnaires assessing motor fatigue are still relevant, especially when it comes to judging the overall benefit of therapeutic interventions and for estimating the prevalence of fatigability in larger patient samples. As a consequence, studies engage both clinical tests and fatigue questionnaires (43).

### Assessment of self-perceived fatigue

Fatigue is reflected to a variable degree by the overall MS-fatigue scores, e.g., the Fatigue Severity Scale and the Modified Fatigue Impact Scale (44, 45). The Fatigue Scale for Motor and Cognitive Function (FSMC) is another well validated instrument for addressing fatigue (15). Based upon FSMC, a recent Norwegian survey among 1,454 patients, found equally high prevalence of motor (82%) and cognitive (72%) fatigue. Despite these already high rates of *subjective motor fatigue*, the prevalence of *objective fatigability* may be even higher since in the absence of subjective fatigue, functional testing may still reveal alterations in motor performance (46). The scores of common fatigue questionnaires correlate with each other, but they may be confounded by general disability and are intended to reflect the multitude of dimensions of fatigue rather than to focus on specific aspects that may be related to pathophysiological changes in exercise responses (10, 47–50).

### Assessment of objective fatigability in response to task performance

From the high prevalence of perceived fatigue in questionnaires one would likewise expect objective fatigability in patients with MS. Although objective fatigability is indeed prevalent in patients perceiving fatigue, the levels of objective and perceived fatigue are only weakly correlated (51). Studies that engaged patients in rather artificial motor tasks, e.g., repeated voluntary contractions of hand- or leg muscles over defined periods and at defined force levels gave conflicting results regarding the correlation of task performance and perceived fatigue scores within the defined scores, although perceived exertion during the task itself was clearly increased in MS-patients (50, 52, 53).

An alternative to the study of fatigability during isolated movements (which are at best fragments of meaningful, intention-guided motor sequences) is testing the patients' performance in more complex tasks which may more closely resemble challenges patients undergo in daily life. One of the most extensively studied and rather easily accessible parameters is walking endurance, defined as the decline of walking speed between the first and the last minute of a 6-min-walking task. Patients with MS show increased objective fatigability in this test when compared to healthy subjects. Furthermore, walking leads to force reductions in distinct muscle groups and to impaired (54).

Besides the retrospectively stated perceived fatigue which is measured in classic fatigue scores (“trait fatigue”) and the objective measurements of functional parameters (e.g., force or velocity), interoceptive signals occurring during physical activity may hamper *ad-hoc* task performance by inducing the feeling of growing exhaustion or difficulty. This so-called “state fatigue” is commonly estimated using visual analog scales during the exercise itself (43). Studies testing muscle force, walking and cognitive tasks could demonstrate clear increases in state fatigue during tasks, but again these increases were only weakly correlated with the objective worsening of performance, i.e., fatigability (55, 56). To explain the fact that classic objective measures of fatigability neither correlate with “state” or “trait”–fatigue, Enoka et al. (43) recently suggested that increasing “extra demands on the nervous system of persons with MS” during task performance lead to fatigue perception. In other words, it is more demanding for MS patients to maintain the nervous drive to activate muscle that is required for movements and maintaining this drive contributes a major part to the perceived fatigability. This hypothesis is in line with previous findings of other groups that studied state fatigue and the effects of training interventions on fatigue parameters, muscle strength and activation parameters (57, 58).

### Assessment of objective fatigability in response to exercise

A critical parameter in the assessment of exercise responses in both healthy and diseased subjects is the duration and the intensity of exercise, the latter usually defined as percentage of the individual's maximum output in that particular task. A special difficulty in MS is that due to the heterogeneity of motor deficits among patients, the results of standard exercise tests show a high degree of variance and are only valid if disability does not interfere directly with engagement in the task, e.g., paresis of the legs with riding on a standard bicycle ergometer or severe ataxia with a simple walking test.

Patients with MS walk slower and their speed declines faster over time than that of healthy controls (59, 60). In contrast, some, although not all studies that assessed isolated muscle fatigability did demonstrate significant differences in force decline during voluntary contractions between MS patients and healthy controls (52, 57, 61). In studies on muscle contractions evoked by peripheral electric stimulation, responses to repeated stimulation have been reported to be reduced in MS patients compared to controls, especially in lower extremity muscles (62–64). Beyond abnormal recruitment responses during voluntary contractions, there clearly is a peripheral component of muscle fatigue that seems to be independent of neurotransmission at the neuromuscular endplate or of sarcolemmnal excitation, since compound motor action potentials are usually unchanged. Nevertheless, the buildup of force during evoked tetanic contractions is reduced and relaxation prolonged (61, 65). Remarkably, also intracellular pH and phosphocreatine have been reported to drop faster in fatiguing muscle of MS patients (65).

From these findings, the question arises whether motor fatigability in MS may be due to insufficient oxygen- or nutrient supply or whether they are caused by changes in neuromuscular structure and function. Before discussing integrative pathophysiological concepts of fatigability in MS, the current knowledge on aerobic capacity as a central component of physical fitness will be summarized.

### Assessing exercise responses of MS patients using spiroergometry

Common measures of physical fitness are derived from parameters measured during spiroergometry challenges. From the analysis of breath gases under and heart rate (maximum heart rate, HRmax), the oxygen uptake rate (VO_2_max or aerobic capacity), the respiratory ratio (RER) and the oxygen uptake efficiency slope (OUES) can be estimated. Oxygen costs for performing daily activities as stair climbing, walking, sitting or standing up or even rolling in bed are higher in MS patients than in age and sex matched controls. The increased oxygen consumption is correlated with higher perceived fatigue (66). This may be the result of less effective movements in MS patients due to altered motor programs. In other words, when compared to healthy controls, MS patients require more energy and thus depend on a better physical fitness to perform equal motor tasks.

Especially the aerobic capacity VO_2_max has widely been used to characterize exercise responses and energy expenditure in MS patients. It is defined as the maximum amount of oxygen an individual can use in a given time and can easily be measured by subtracting the amount of oxygen in the inspired from that in expired air. A strong correlation exists between an individual's VO_2_max and its ability to engage in endurance motor tasks, but also in many other kinds of physical activities (67, 68). Aerobic capacity is thus not identical with physical fitness, but besides strength, flexibility and other parameters it is one of its central components.

To be extracted from the inspired air, oxygen has to be utilized by working muscle or other tissues. In healthy humans, the amount of oxygen which would be utilized if all muscles were intensely activated at the same time by far exceeds the amount of oxygen that can be delivered to them by the cardiovascular system. Therefore, it is the capacity of the cardiovascular system to deliver oxygen that sets the upper limit for aerobic endurance performance in motor tasks. In neurological disorders, however, the activation of muscles and therefore their cumulative oxygen utilization may be restricted. In such a situation, which may also occur in MS, a reduced VO_2_max may reflect limitations of physical activity by disability itself rather than the limitations of the cardiovascular system response. Therefore, it is of critical importance to apply the rather strict criteria for the estimation of VO_2_max that have been introduced by Midgley et al (69) and which were applied in the studies by Langeskov–Christensen et al. (73, 79). These require that

1. The measured O_2_-uptake remains constant despite increasing workload.
2. The achieved heart rate is close to the expected heart rate calculated from the individual's age,
3. The measured RER is above 1.1 and that the subjective rating of exertion exceeds predefined values (e.g., Borg's rating of perceived exertion > 16).

Fulfillment of criterion 1 means that the individual is shifting to anaerobic metabolism to provide energy for the increasing muscle work, since no additional O_2_ can be delivered. Criterion 2 relies on the fact that oxygen consumption depends on oxygen transport through the circulation and thus cardiac output, as reflected by the strict correlation between HRmax and VO_2_max (68). Criterion 3 means that the amount of CO_2_ that is expired per time is above that of inspired O_2_. The additional release of CO_2_ from plasma bicarbonate stores is reflects the acidification of the blood during anaerobic metabolism, i.e., lactic acidosis [criterion 1, (70)].

Spiroergometry has been thoroughly validated for the use among ambulant MS patients (71–73) and used extensively to study the effects of training and other interventions on MS patients' physical fitness (74). A systematic review and meta-analysis identified 40 studies that altogether analyzed data of 1,029 MS patients and 165 healthy controls (73). When comparing the results of classic whole-body spiroergometry, the mean value reported in studies was 25.2 ± 5.2 ml·kg^−1^· min^−1^ for MS patients and 30.9 ± 5.4 ml·kg^−1^·min^−1^ for controls. For spiroergometry restricted to upper limb muscles, the respective values of the single study (75) that compared both groups, the respective values were 10.2 ± 4.7 ml·kg^−1^·min^−1^ (MS patients) vs. 14.3 ± 1.6 ml·kg^−1^·min^−1^ (controls).

As a result of their 2015 meta-analysis, Langeskov-Christensen et al. (73) report a significant reduction of the VO_2_max among MS patients over the pooled sample. Three of the five included studies reported significantly lower VO_2_max values among MS patients compared to controls (76–81). Lower mean values of VO_2_max in the studied patient samples correlated with higher mean disability and increased age (73).

Additional studies published after the abovementioned meta-analysis added further evidence to support the relevance of reduced aerobic capacity in MS. The work of Klaren et al. included 162 MS patients and 80 controls and reported significantly lower values for VO_2_max, RER, HRmax and other parameters among MS patients. Furthermore, when MS patients were classified according to their scores on the patient determined disease steps (PDDS)–scale, significant differences in VO_2_max could be observed between those defined to have mild, moderate and severe disability, with the lowest values seen in the group with the highest degree of disability (82). Likewise, a study by Driehuis et al. found reduced VO_2_max in MS patients compared to reference values. However, in the studied sample there was no correlation between VO_2_max and physical activity. A correlation with fatigue as measured by the “Checklist for Individual Strength 20r” was reported by the authors (83) whereas two other recent studies and another meta-analysis reported only weak or even lacking correlations between reductions of VO_2_max and fatigue (84–86).

Taken together, different independent studies indicate that VO_2_max is reduced in MS. Although the relevance of this reduction in aerobic capacity is less clear we will subsequently discuss their pathophysiology in the context of reduced physical activity and autonomic function.

### Pathophysiological concepts of reduced aerobic capacity in MS

**Figure 1:**
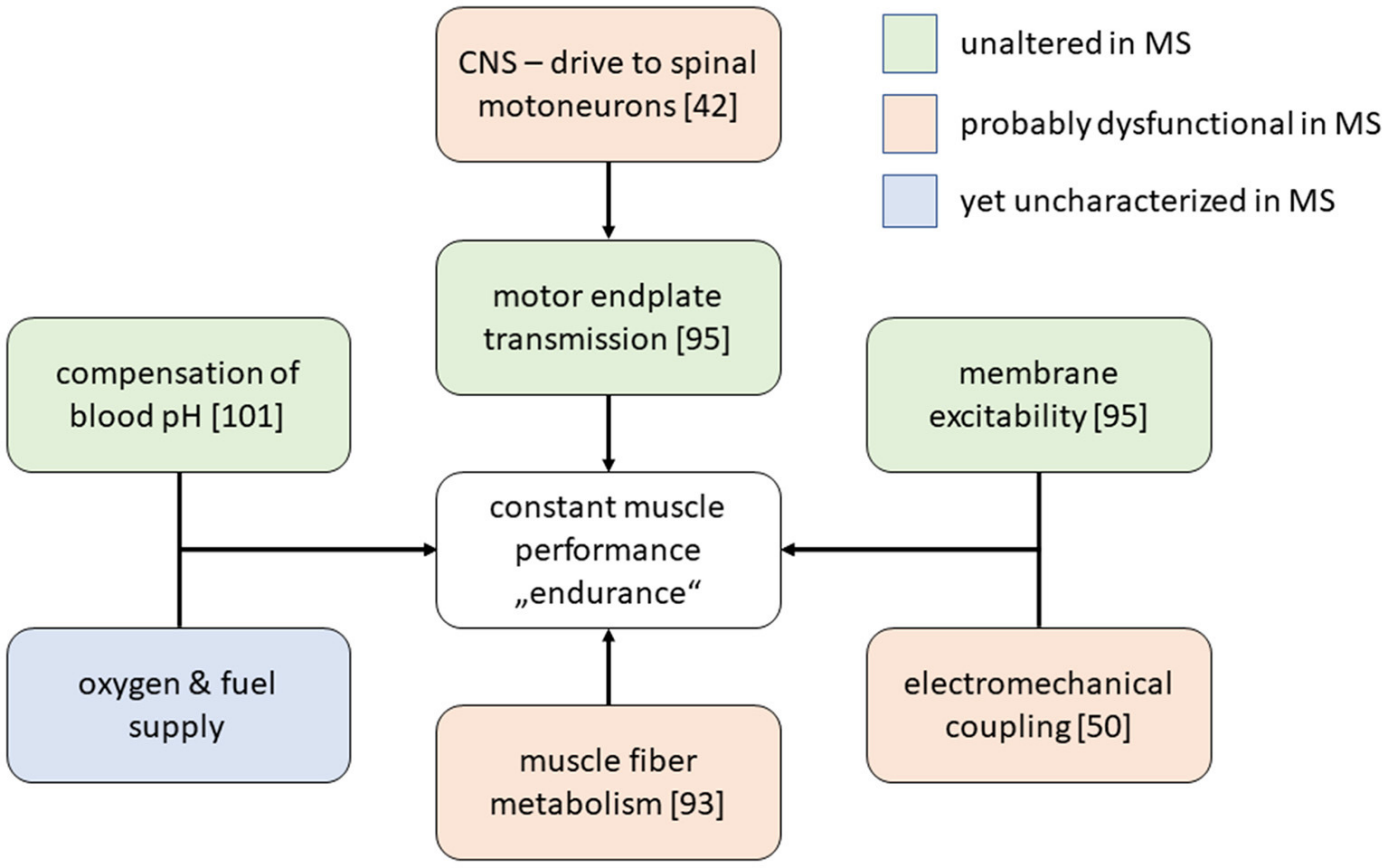
Selected physiological processes that are required to maintain stable muscle force production. Among MS-patients, most of these processes have been described in MS patients samples and, according to the given in the figure legend, were found to be altered or not. The metabolic integration between muscle, liver and other organs during exercise has not yet been characterized thoroughly by appropriate experiments in MS patients.

Basically, two different and at least partly conflicting pathophysiological concepts of MS-related limitations in cardiorespiratory parameters exist and will be discussed here. First, the mere lack in physical activity may be considered the central or even the only causative factor. We will thus refer to this concept as the *deconditioning hypothesis*. Second, CNS-lesions causing alterations in autonomic function on the level of cardiac, respiratory and vascular control can be considered to hamper the appropriate physiological adaptive responses during exercise, a concept which may be termed the *central dysregulation hypothesis*. Despite the fact that a combination of both seems likely to appear in reality, a dominance of one of the components is suggested by some authors (87). A brief summary of the involved factors is given in Figure 1.

In more detail, the deconditioning hypothesis implies that reduced VO_2_max is caused by a decreased metabolic activity of contracting skeletal muscle, i.e., an alteration of muscle metabolism that cannot be explained by an acute innervation deficit of the activated muscle. Instead it is suggested that the chronic lack of exercise and muscle activation would cause a loss in the oxidative capacity of muscle fibers due to altered mitochondrial function, thereby reducing oxygen utilization (87). However, even under regular physiological conditions, a considerable part of skeletal muscle fibers is not capable of a fully oxidative metabolism and releases lactate to the blood which is either metabolized by other tissues or converted to glucose by the liver and then returned back to the muscle (88, 89). As a consequence, oxygen utilization by the liver increases during exercise in healthy subjects (90–92).

To our best knowledge, despite the well-known associations between altered liver function and fatigue in other medical conditions, there are no studies addressing hepatic function during exercise among MS patients.

In contrast, as already stated above, alterations of muscle function in MS have been described even decades ago on the level of altered contraction dynamics and muscle architecture and strength (93, 94). In addition to that, fibers of the tibialis anterior muscle from MS patients are smaller, have an impaired oxidative capacity (which is frequently interpreted as impaired mitochondrial function) and rely more on anaerobic metabolism than those obtained from healthy controls (95, 96), but myosin-ATPase activity is not increased in MS patients compared to controls (97). This finding indicates that fatigability during exercise in MS patients is unlikely to be the result of increased energy demands of the activated muscle. Within the deconditioning hypothesis, this is an important complementary information since it allows the conclusion that the reduced VO_2_max is indeed reflecting reduced muscle activity. Otherwise, it could be argued that increased energy demands of rather few activated fibers might lead to local metabolic decompensation and rapid exhaustion despite a globally decreased energy and oxygen consumption. Functional measurements by Kent-Braun et al. (98) indeed gave no evidence for metabolic failure in contracting muscles of MS patients. Whereas, deconditioning can be expected to be prominent in the leg muscles of MS patients with impaired ambulation, one would not expect it to occur in non-affected upper extremity muscles. Thus, the results of two recent studies that demonstrated reduced oxidative capacity in both leg (gastrocnemius) and wrist flexor muscles of MS patients compared to controls raise a challenge to the deconditioning hypothesis (99, 100).

The central dysregulation hypothesis assumes that the limited ability of MS patients to exercise is impaired due to altered cardiac, circulatory, respiratory or thermoregulatory responses (101).

Whereas, cardiac and circulatory responses in MS have been addressed by various studies, rather few have covered respiratory and thermoregulatory responses. On average, pulmonary function in terms of spirometry responses is not altered in MS, although individual patients may show signs of impaired respiratory muscle strength (102). During exercise, ventilatory dysfunction has been reported in MS patients, i.e., the efficiency of ventilation seems to be rather low and could not be improved by a 6-month training intervention (103). The relevance of this finding with respect to fatigability still remains to be defined. At least, lactate levels do not differ significantly between MS patients and controls during exercise, indicating that there is probably no increased demand for respiratory compensation to avoid relevant pH-shift (104).

The sweating response to exercise is impaired in MS patients due to impaired sudomotor function, leading to larger increases in body temperature during exercise with potential detrimental effects on performance. Results of studies that aimed to quantify this effect gave conflicting results (105–108).

The pathophysiological link between cardiovascular dysregulation and impaired exercise performance is rather straightforward. Briefly, the insufficient cardiac inotropy or chronotropy would prevent the necessary increase in cardiac output to permit delivery of dissolved oxygen to the tissues. On the other hand, a failing vasomotor response may lead to an inadequate allocation of cardiac output with a relative perfusion deficit in working muscle or to systemic hypo- or hypertension during exercise. Clinical data point to a rather high prevalence of autonomic dysfunction in MS (109). In particular, cardiac function seems to be altered in MS patients compared to controls and even severe neurogenic cardiomyopathy has been reported to occur (110, 111). Heart rate variability is an easily available parameter that reflects cardiac autonomic control and indeed gives pathological findings in MS patients, with a majority of studies indicating a correlation with CNS lesions in regions that are associated with autonomic regulation of cardiac rhythm (112–116). In studies investigating exercise effects on aerobic capacity of MS patients, abnormal heart rate responses were reported which in some studies were ameliorated to training, whereas they abnormal in others (87, 117, 118). Another recent finding is that the recovery of heart rate following exercise *via* parasympathetic pathways is impaired in MS patients. This is in line with other data indicating parasympathetic dysfunction in MS (100, 101). Besides heart rate modulation, an increase in stroke volume is a regular response to exercise. This response was shown to be diminished in a cross-sectional study comparing MS patients and controls and was not reversed by a 6-month-training intervention. Instead, MS patients keep their systemic blood pressure by increasing vascular resistance with potential negative effects on cardiac workload (119, 120).

Although the evidence suggests that both deconditioning and autonomic dysfunction are frequent in MS and may even potentiate each other, it is rather difficult to specify their contribution to clinical fatigability or perceived fatigue in individual patients. Neither aerobic capacity nor autonomic dysfunction are strongly–if at all–correlated with classic fatigue scores or quality of life (85, 86, 121, 122).

### Training effects on cardiorespiratory fitness

The question whether it is possible to raise the reduced VO_2_max in MS patients by training is relevant in the light of the discussion whether altered the cardiorespiratory fitness of MS patients is a consequence of autonomic dysregulation or other primary sequela of the immune mediated disease, or whether it is merely a consequence of deconditioning and lack of exercise.

In contrast to earlier recommendations to restrict physical activity in MS patients, cardiorespiratory fitness in patients with MS can be increased safely and effectively by appropriate training and exercise (123). Endurance- and resistance training of moderate intensity are recommended for patients with mild to moderate disability (124). Classic types of endurance training are bicycle ergometry, combined arm-leg or isolated arm ergometry and treadmill walking. Individual circumstances should nevertheless be considered: Patients receiving oral or intravenous glucocorticosteroids are at an increased risk for acute hypertension and hyperglycemia, so a monitoring of these parameters is recommended before and during exercise. Relevant critical events or severe deteriorations of mental or physical health have hardly ever been reported to occur in physical exercise programs for multiple sclerosis and relapses do not occur more frequently in MS patients on exercise programs. An instructive review on practical aspects of exercise training in MS is given by Learmout and Motl (125).

Beneficial effects on fatigue scores and QoL can be achieved for patients participating structured training programs (122, 126–128). Aerobic exercise has been especially well-studied with this respect. In their recent meta-analysis, Andreu-Caravaca et al. analyzed 43 studies that had investigated effects of aerobic training or control interventions on functional parameter (i.e., walking speed and endurance) as well as on parameters reflecting cardiorespiratory fitness (i.e., VO_2_max). In the meta-analysis, significance for improved cardiorespiratory fitness could only be demonstrated for interventions that applied moderate intensity bicycle training at least 3 days a week on moderate intensity. Furthermore, most likely due to heterogeneity in study protocols and studied patient samples, the pooled analysis of studies could not detect a difference between aerobic training and control interventions, most of which applied some kind of exercise training as well.

As already mentioned, the extent of improvement in cardiorespiratory fitness varied widely, depending on the applied interventions: Whereas, for instance, a study by Mostert and Kesselring (129) found no increase in VO_2_max following a rather short intervention period of 3 weeks with 30 min of training for 5 days a week, Ponichtera-Mulcare et al. (130) and Rodgers et al. (131) reported an almost 20%-increase in ambulatory, but of only 5% in non-ambulatory MS-patients following a 6-month intervention of aerobic exercise on every second day for 30 min.

Whereas, physical activity is usually beneficial when conducted in a safe framework and at an individually optimized intensity, some patients may report even increased fatigue or a worsening of other symptoms. To improve the applicability of aerobic training in such patients, special modifications have been developed to avoid potential detrimental effects of training. Increases in body temperature during training which might lead to a worsening of MS symptoms in predisposed patients are prevented in special aquatic exercise programs. Studies that investigated the effects of exercise while immersed in water (usually 28°C) found beneficial effects on QoL and fatigue (132, 133) as well as on cardiorespiratory fitness (134). Furthermore, to achieve larger effects on endurance with lesser intensity of training, e.g., in patients that have severe fatigability, normobaric hypoxic endurance training might be an alternative strategy since it takes advantage of the same physiological mechanisms that are applied in high altitude training (135, 136), although by now, none of the studies investigating hypoxic endurance training could prove superiority to standard exercise. Treadmill walking and strength training seem to be less suitable to increase cardiorespiratory fitness (137). A very recent meta-analysis concluded that combined endurance and resistance exercise programs have the highest probability to improve both subjective fatigue and objective fatigability (138). Besides potential positive effects of physical activity on cardiovascular fitness and fatigue, patients may be encouraged to participate in other exercise programs with potential benefits for quality of life, e.g., by improving bladder control (139).

Taken together, the existing literature gives evidence that training interventions of appropriate duration and intensity can increase cardiorespiratory fitness in MS patients. However, even with sophisticated and well-instructed interventions of 6-month training, the reported VO_2_max values of MS-patients are clearly lower than those reported for general population samples (e.g., median values for 50-50 year old males: 38.5 ml·kg^−1^·min^−1^; females: 31.0 ml·kg^−1^·min^−1^) (140). Compared to these general reference values, the *ad-hoc* VO_2_max of healthy controls that are reported in the literature appear rather low. To our knowledge, there are no studies that have directly compared the training-induced increases in VO_2_max of healthy controls and MS patients, which would be helpful to answer the question whether detraining or MS itself contribute more to the impaired cardiorespiratory fitness of MS patients.

## Summary and conclusion

Motor fatigue is a frequent and disabling symptom of MS. It can be assessed using questionnaires that in general assess the perceived quality, intensity and temporal aspects in a retrospective manner (“trait fatigue”). In contrast, the perceived exhaustion during motor task performance or at other defined time points can be estimated using instantaneous ratings, e.g., *via* visual analog scales (“state fatigue”). Objective functional measurements, e.g., walking distance or force generation clearly demonstrate change of performance indicating fatigability in MS patients. These are, however, not strictly correlated with perceived state or trait fatigue. Physical disability that is related to the primary CNS lesions in MS, e.g., paresis and ataxia, have a major influence on fatigue parameters and constitute methodological problems for defining the pathophysiology of the observed phenomena since they overlay with prominent secondary factors as deconditioning of peripheral muscle and autonomic reflexes as well as with other more subtle primary CNS-related sequelae, e.g., damage to autonomic regulatory pathways or complex cortical networks involved in motor planning and interoception. As a result of combined deconditioning and altered autonomic function including pathological cardiovascular function, the aerobic capacity is clearly reduced in MS-patients which inevitably reduces physical fitness, although the observed degree of correlation between the degree of impaired aerobic capacity varies widely between studies, most likely due to confounding effects of general disability and other factors affecting trait fatigue. Based upon the hypothesis that deconditioning due to a deficit in physical activity is a major factor in the pathogenesis of motor fatigue, training interventions have been extensively studied and have been shown to be safe and effective for improving physical fitness in MS-patients. Training effects on fatigue vary widely between studies and again depend on the patient's disability, comorbidities and on the applied training protocol. An integrated and personalized approach is thus necessary for addressing motor fatigue in MS patients.

## Author contributions

RP and UZ conceptualized the study, collected and discussed the literature, and prepared the manuscript. Both authors approved the final version.

## Conflict of interest

Author RP has received research grants from Novartis. Author UZ received research support as well as speaking fees and travel funds from Almirall, Alexion, Bayer HealthCare, Bristol Myers Squibb, Biogen, Janssen, Merck Serono, Novartis, Octapharma, Roche, Sanofi Genzyme, and Teva.

## Publisher's note

All claims expressed in this article are solely those of the authors and do not necessarily represent those of their affiliated organizations, or those of the publisher, the editors and the reviewers. Any product that may be evaluated in this article, or claim that may be made by its manufacturer, is not guaranteed or endorsed by the publisher.

## Abbreviations

CNS: Central Nervous System
EDSS: Expanded Disability Status Scale
MS: Multiple Sclerosis
OUES: Oxygen Uptake Efficiency Slope
VO_2_max: maximum oxygen uptake
VO_2_peak: peak oxygen uptake.
